# High Quality Factor, High Sensitivity Metamaterial Graphene—Perfect Absorber Based on Critical Coupling Theory and Impedance Matching

**DOI:** 10.3390/nano10010095

**Published:** 2020-01-02

**Authors:** Chunlian Cen, Zeqiang Chen, Danyang Xu, Liying Jiang, Xifang Chen, Zao Yi, Pinghui Wu, Gongfa Li, Yougen Yi

**Affiliations:** 1 Joint Laboratory for Extreme Conditions Matter Properties, Southwest University of Science and Technology, Mianyang 621010, China; cenchunlian@mails.swust.edu.cn (C.C.); JLY18181124563@yeah.net (L.J.); chenxifang1988@yeah.net (X.C.); 2 Research Center for Photonic Technology, Fujian Key Laboratory for Advanced Micro-nano Photonics Technology and Devices & Key Laboratory of Information Functional Material for Fujian Higher Education, Quanzhou Normal University, Quanzhou 362000, China; czqchem@qztc.edu.cn; 3 College of Science, Zhejiang University of Technology, Hangzhou 310023, China; xudanyang@zjut.edu.cn; 4 Key Laboratory of Metallurgical Equipment and Control Technology of Ministry of Education, Wuhan University of Science and Technology, Wuhan 430081, China; ligongfa@wust.edu.cn; 5 College of Physics and Electronics, Central South University, Changsha 410083, China; yougenyi@csu.edu.cn

**Keywords:** metamaterial, graphene, critical coupling, perfect absorption, high quality factor

## Abstract

By means of critical coupling and impedance matching theory, we have numerically simulated the perfect absorption of monolayer graphene. Through the critical coupling effect and impedance matching, we studied a perfect single-band absorption of the monolayer graphene and obtained high quality factor (Q-factor = 664.2) absorption spectrum which has an absorbance close to 100% in the near infrared region. The position of the absorption spectrum can be adjusted by changing the ratio between the radii of the elliptic cylinder air hole and the structural period. The sensitivity of the absorber can be achieved S = 342.7 nm/RIU (RIU is the per refractive index unit) and FOM = 199.2 (FOM is the figure of merit), which has great potential for development on biosensors. We believe that our research will have good application prospects in graphene photonic devices and optoelectronic devices.

## 1. Introduction

In recent years, plasmon metamaterial have attracted extensive attention because of their unique EM (electromagnetic) control ability [1,2,3,4]. Because of its physical and optical properties, it has become the most popular material for studying. At first, electromagnetic metamaterials could only be used in the microwave frequency range, but as people continue to study, their applications range from terahertz to infrared and then to almost the entire electromagnetic spectrum of visible light [5,6,7]. Meanwhile, the ability of metamaterial absorbers to enhance absorption in the microwave, infrared, visible, and solar systems has also been demonstrated. Metamaterials have great potential in the control of light polarization, phase, and amplitude, which can be applied in optoelectronics, photonics, and photocatalysis [8,9,10,11,12,13,14,15,16,17,18,19]. Due to its super control of light, some new components can be developed, such as broadband circular polarizer, new perfect absorber and so on.

Graphene, as a new type of two-dimensional material that has great research value in optical and electronic properties [20,21,22,23]. Graphene has extremely high carrier mobility [24,25] and full spectral response to light in the ultraviolet to terahertz band, and ultra-fast response to light, making it a perfect optoelectronic device material. The application of levitated monolayer graphene in photovoltaic field is limited to some extent because its absorption efficiency of ordinary incident light is merely 2.3% [26]. All in all, resonance effect is the key method for enhancing material absorption or emission [27,28]. In the middle and far infrared bands, graphene has high plasma resonance, which is widely made use of enhancing the absorption efficiency of graphene. Coupling of graphene to a dielectric or metal resonant structure typically enhances the absorption of graphene under visible and near-infrared conditions. However, graphene-based high-performance optoelectronic devices have higher requirements for high absorption, such as requiring good mechanical flexibility, fatigue stability, excellent electrochemical and thermal stability, etc. [29], and pure monolayer graphene cannot achieve complete absorption due to metal decay and surface reflection. Therefore, in the optical range, achieving perfect absorption based on a monolayer graphene structure remains a huge problem.

Based on the above inspirations, we numerically simulated the perfect absorption of monolayer graphene using impedance matching theory and critical coupling. We studied the perfect absorption of monolayer graphene in a single band in the near-infrared region through using the critical coupling theory, and obtained high quality factor band absorption spectrum with an absorbance of nearly 100% in the resonance wavelength region. In this research, the phenomenon that critical coupling can enhance perfect absorption of graphene can be understood through coupling mode theory (CMT) and impedance matching. The optical properties of the whole structure can be calculated by The Finite Difference Time Domain (FDTD) method. By changing the thickness of polymethyl methacrylate (PMMA) and the dielectric layer, we can control the changes of the absorption efficiency of graphene at greatly different working wavelengths. In addition, the relationship between the radii of the elliptical cylindrical air hole to the period of the structure and the sound absorption performance was studied. Finally, the sensing performance of such perfect absorbers in the refractive index sensor was studied. On the basis of graphene, this method lays a solid theoretical foundation for the research of photonic devices and optoelectronic devices.

## 2. Structure and Theory

**Figure 1:**
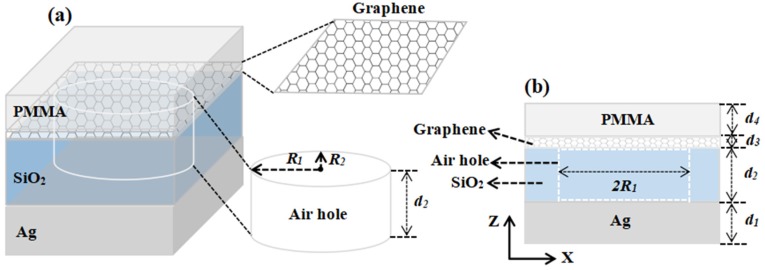
(**a**) Schematic diagram of the three-dimensional structure based on a monolayer graphene perfect absorber. (**b**) Design the *x*-*z* section view of the structure.

Figure 1a presents a three-dimensional structure diagram of a perfect absorber for monolayer graphene. The silicon dioxide (SiO_2_) layer has a periodic elliptic cylinder air hole and monolayer graphene between it and the top layer of two-dimensional polymethyl methacrylate (PMMA), and the silver (Ag) layers are stacked on the floor of the entire structure and act as the back of the mirror to block the direction of light propagation. The specific structural geometry parameters are that the thickness of Ag, SiO_2_ layers are set as *d*_1_ = 275 nm, *d*_2_ = 235 nm, the thickness of elliptical cylindrical air hole and the radii of long and short axis are taken to be *d*_2_ = 235 nm, *R*_1_ = 440 nm, *R*_2_ = 380 nm, the thickness of graphene monolayer and PMMA layer are *d*_3_ = 0.34 nm and *d*_4_ = 50 nm, respectively, and the structure’s period is *P* = 1000 nm. In the near-infrared area, the refractive index data used for graphene can be fitted by the formula n=3+j5.446λ/3 [30]. Throughout the simulation calculations, except for special instructions, we study the TM polarization (electric polarization parallel to the *X*-axis) light shining onto the entire system at the condition of normal incidence. The *X*-*Z* section of the designed structure as shown in Figure 1b. The refractive indices of air, SiO_2_ and PMMA are 1, 1.45, and 1.48, respectively. The numerical simulation results are analyzed by using Lumerical Solutions software and the Finite Difference Time Domain (FDTD) method [31,32]. The metal mirror material is Ag, and its relative dielectric constant [33] in the visible light band can be given by the Drude model [34,35,36]:

(1) $$\varepsilon (\omega )=\varepsilon_{\infty }-\frac{\omega_{p}^{2}}{\left(\omega^{2}+i\gamma \omega \right)}.$$

For Ag, these physical parameters can be set to *ε*_∞_ = 3.7, *ω_p_* = 1.38 × 10^16^ s^−1^, *γ* = 2.73 × 10^13^ s^−1^ [37,38].

In this structure, the transmission of incident light can’t be realized because of the influence of photonic band gap effect, and its absorption rate can be expressed by CMT [39,40,41,42]:

(2) $$A=1-\Gamma (\omega )=\frac{4\delta \gamma_{e}}{{\left(\omega -\omega_{0}\right)}^{2}+{\left(\delta +\gamma_{e}\right)}^{2}}.$$

In the above formula, *ω*_0_ is the resonance angular frequency, *δ* and *γ_e_* are referred as internal loss and external leakage rate. It can be obtained from Equations (2) that when the resonance state (*ω* = *ω*_0_) occurs, the external leakage rate is equal to the internal loss rate of graphene (*γ_e_* = *δ*). At this time, the critical coupling condition is completely satisfied, and the reflection coefficient is gone, and all incident energy is absorbed, and the absorption rate can reach 100%. This situation is called critical coupling.

The virtual impedance about a perfect absorber can be expressed as [43,44,45]:

(3) $$Z=\sqrt{\frac{{\left(1+S_{11}\right)}^{2}-S_{21}^{2}}{{\left(1-S_{11}\right)}^{2}-S_{21}^{2}}}.$$

In the formula (3), *S*_11_ is the scattering parameters and *S*_21_ is the transmission coefficients, which are closely related to the reflectivity. In fact, the system is considered as a simplex port system (|*S*_21_| = 0), because the photonic band gap effect of photonic crystals will seriously hinder its transmission. In order to effectively suppress the reflection of the system (|*S*_11_| = 0), it is necessary to satisfy the virtual impedance about the system and the free room impedance in the resonant wavelength region. Therefore, we know that the system can completely absorb the incident energy. Moreover, in our simulation calculation, *x* and *z* directions are specified as periodic boundary conditions and absorption boundary conditions of exactly matching layer, respectively.

## 3. Results and Discussions

**Figure 2:**
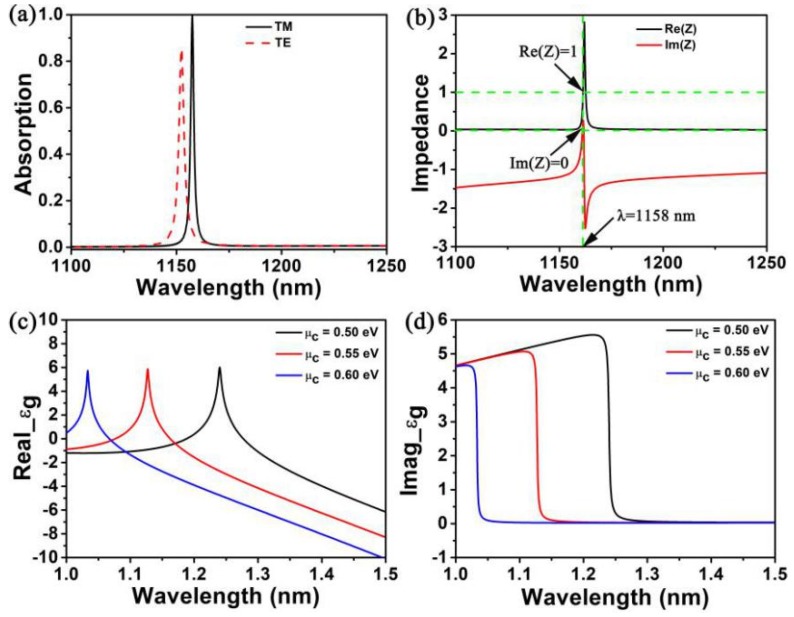
(**a**) The absorption spectra about the transverse magnetic (TM) and transverse electric (TE) polarizations of the structure. (**b**) The real and imaginary parts of the virtual impedance (*Z*) about the perfect absorption peak are black line and solid line respectively. (**c**) The real and imaginary parts of monolayer graphene. (**d**) Functional relation of permittivity with chemical potential (*μ_c_*).

In Figure 2a, we studied the polarization of TM (transverse magnetic, the electric field is parallel to the *X* direction) and TE (transverse electric, the electric field is parallel to the *Y* direction) polarized absorption spectra with the same geometrical dimensions of the structure. According to the figure, the absorption of TM polarization at a wavelength of 1158 nm reaches about 100%, while the TE polarization has the same geometrical size of the structure, the absorption peak wavelength shifts to the left, and the absorption peak wavelength is 1152 nm. The absorption rate is about 85.5%. The reason is the proposed elliptic cylindrical structure is not highly symmetrical, which results in the difference of absorption peak wavelength and absorption rate between TM and TE polarization. According to Figure 2b, when the resonant wavelength is 1158 nm, the virtual impedance of the entire structure is exactly matched with the free room impedance (*Zω*_0_ = 1) and the whole structure is fully absorbed. The rationale is that minimizing the reflectivity by adjusting the degree of matching between free room and impedance (*Z* = 1), and for the purpose of removing the transmittance of light by using a metal mirror which has a definite thickness. Figure 2c,d show the functional relationship between the permittivity of real and imaginary parts of monolayer graphene with chemical potential (*μ_c_*) [46].

**Figure 3:**
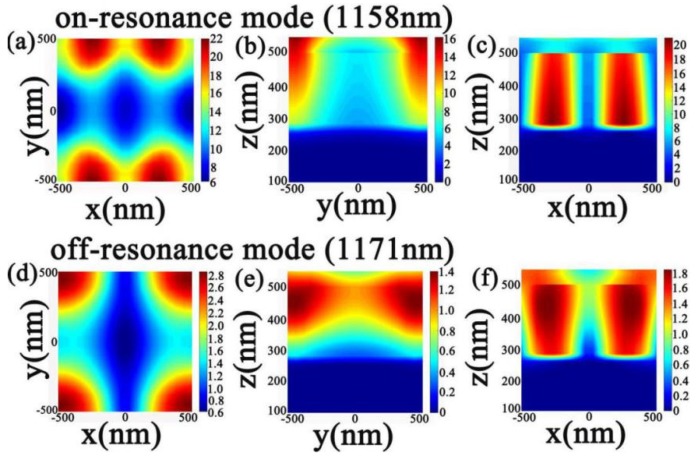
Simulates the electric field (|*E*|) distribution of (**a**–**c**) resonance modes (1158 nm) at *x*-*y*, *y*-*z*, and *x*-*z* based on graphene structure under normal incidence and (**d**–**f**) are the electric field (|*E*|) distributions of *x*-*y*, *y*-*z* and *x*-*z* in the non-resonant mode (1171 nm).

For the purpose of deeper proof of the efficient absorption response about monolayer graphene under normal incident conditions, we present the distribution of electric fields (|*E*|) in *x*-*y*, *y*-*z*, and *x*-*z* in the resonant mode (1158 nm) and non-resonant mode (1171 nm) based on the graphene structure in Figure 3a–c and Figure 3d,e, respectively. As shown in Figure 3a–f, in the critical coupling state, when the cavity is triggered (resonant mode), the absorption peak (1158 nm) of the black curve in Figure 2a appears. At this time, the electric field intensity is arranged as Figure 3a–c. It can be seen from the figure which has a strong electric field around the graphene. If the cavity is in an unexcited state (non-resonant mode), we can find that the reflection coefficient about the lossless system is −1, which is the low absorption value (1171 nm) of the black curve in Figure 2a, and Figure 3d,e is the distribution of electric field intensity. For the upper layer of the whole system, the electric field distribution in *x*-*y* direction shows that at the wavelength of 1158 nm, the electric field is principally concentrated around the graphene of the cavity. For the electromagnetic field at the central position, it is well consumed by graphene due to resonance, which is it is absorbed by graphene. However, for the *x*-*y* direction at the non-resonance (1171 nm), the incident light field in the resonant cavity at the *x*-direction is not completely consumed, so the absorption performance of the system at the non-resonance is lower than that at the resonance mode. For the same reason, the same characteristics are obtained for the resonance and non-resonance modes in the *y*-*z* and *x*-*z* directions.

**Figure 4:**
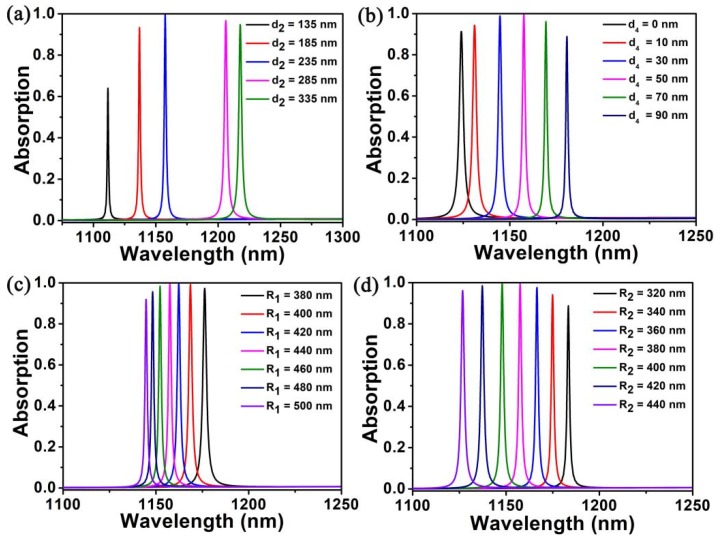
(**a**) Absorption spectra of different SiO_2_ (*d*_2_) thicknesses; (**b**) Absorption spectra with the different thicknesses of PMMA (*d*_3_); (**c**) Absorption spectra of the long axis radii (*R*_1_) of different elliptical cylindrical air hole; (**d**) Absorption spectra of various short axis radii (*R*_2_) of elliptical cylindrical air hole.

Since the absorption factor about the monolayer graphene has nearly no relation to frequency in the light wave range of our experiment, which also has a stable internal loss rate (*δ*), and it can be concluded that the perfect absorption of graphene depends on the external leakage rate (*γ_e_*) which can control its structure. In addition, we have also done some research on changing the relevant parameters to affect the absorption rate of graphene, as well as the relationship between various structural parameters and external leakage rate. As the thickness of SiO_2_ (*d*_2_) and PMMA (*d*_4_) increases, we can clearly see the absorption peak wavelength of monolayer graphene experienced a red shift, meanwhile, the absorption peak first increased and then decreased, as shown in Figure 4a,b. The main reason for this phenomenon is that the system experiences three status of under-coupling, critical coupling, and over-coupling with the leakage rate of resonance increasing. The spectral lines of *d*_2_ = 235 nm and *d*_4_ = 50 nm in the figure are the perfect absorption of the single layer graphene, which only occurs in the critical coupling stage. Through changing the thickness of SiO_2_ and PMMA, the spectral selectivity of the model structure will also be increased, which provides experimental feasibility for the design in this chapter. In addition, we also studied the influence of the radii of the elliptical cylindrical air hole on the absorption performance of monolayer graphene structure. Equalizing the external leakage rate to the inherent loss rate of the guided mode resonance is the key to achieving the critical coupling state, and the monolayer graphene is the main factor which causes the intrinsic loss. In the photonic crystal plate, the leakage rate of guide mode resonance is mainly determined by the ratio of stomatal radii of the elliptic cylinder air hole and its period, which is R/P. Due to the intrinsic loss rate of the graphene monolayer is not related to the radii, when the period is unchanged, the external leakage rate is proportional to stomatal radii of the elliptical cylindrical air hole and increases with it. At the same time, it is found that perfect absorption and critical coupling of the stomatal radii of the elliptical cylindrical air hole are generated at *R*_1_ = 440 nm and *R*_2_ = 380 nm. Therefore, the critical coupling can be achieved more conveniently only by properly designing the ratio of radii to the period of the elliptical cylindrical air hole. With the increase of the long axis (*R*_1_) and the short axis radii (*R*_2_) of the elliptical cylindrical air hole, the wavelength of the absorption peak experienced blue shift, as shown in Figure 4c,d. The reason is the area of the elliptical cylindrical air hole increases with the increase of the radii, which results in the decline of the effective refractive index of the guide mode resonances.

**Figure 5:**
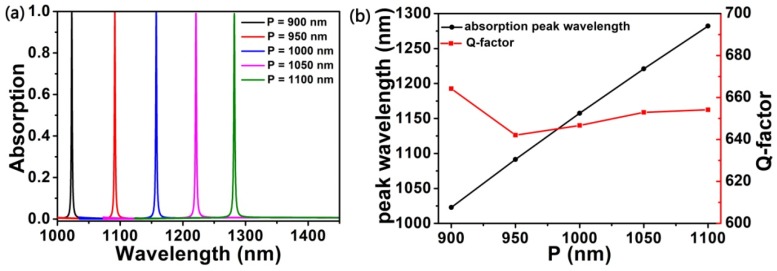
(**a**) Absorption spectra based on different periods (P) of graphene structure. (**b**) Functional relation between period change and wavelength of absorption peak and Q-factor.

Once the ratio between radii and period of the air hole of elliptic cylindrical is determined and the critical coupling is achieved, the wavelength of the absorption peak can be red shifted regularly by increasing the period value of the structure, as shown in Figure 5a,b black curve. When the periods are 900 nm, 950 nm, 1000 nm, 1050 nm, and 1100 nm, the corresponding perfect absorption peak wavelengths are 1022.9 nm, 1091.5 nm, 1157.6 nm, 1220.9 nm, and 1282.1 nm, respectively. We further studied the quality factor (Q-factor = λ/FWHM) of the structure and found that with the increase of the period (P), the Q-factor first decreased and then gradually increased, but the Q-factor basically remained above 640. When the period is 900 nm, the maximum Q-factor corresponding to the model is 664.19. When the periods are 900 nm, 950 nm, 1000 nm, 1050 nm, and 1100 nm, the corresponding Q-factor are 664.2, 642.1, 646.7, 652.9 and 654.2, respectively, as shown by the red curve in Figure 5b. In order to evaluate the performance excellence of proposed structure, we compared the results with other similar structures listed in Table 1 based on the period (P) is 900 nm. Obviously, our structure exhibits high quality factor. Compared with the reported research results, we proposed absorber has a relatively high Q-factor [34,42,47,48,49,50]. Therefore, this perfect absorption system has good tenability and has important applications in nanoscale wavelength division multiplexing metamaterial absorber. At the same time, this conclusion has important guiding significance for experimental design.

The proposed monolayer graphene structure can be used to measure the refractive index of the metamaterial surface environment, and it has the advantages of narrow band width and large modulation depth. In the actual working environment, when the refractive index of the metamaterial of the local surface isoexcimer resonance sensor changes with the incident wavelength, the light intensity can usually be measured [51,52,53,54,55,56,57]. Therefore, we propose the definitions of sensitivity (*S*) and figure of merit (*FOM*), so as to prove the sensor performance of the studied structure, as shown below [58,59,60,61,62,63,64,65,66,67,68]:

(4) $$S=\frac{\Delta \lambda }{\Delta n},$$

(5) $$FOM=\frac{S}{FWHM}.$$

**Figure 6:**
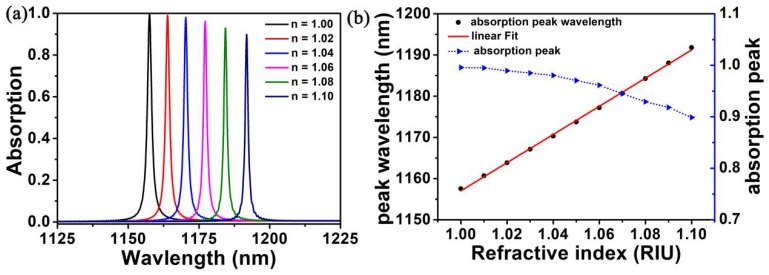
(**a**) The absorption spectra on the basis of refractive index (n) about the surrounding medium with different graphene structure. (**b**) Functional relation between refractive index change and wavelength of absorption peak (red line) and fitting relation (blue line).

Figure 6a shows that when the refractive index of the nearby medium changes from 1.00 to 1.10 (each change is 0.02), the wavelength of the resonance peak shifts red. The absorption rate at the resonant wavelength is as high as 0.90, and the full width at half maximum (FWHM) is narrower than 1.09 nm. With the refractive index improving, the resonance wavelength shifted from 1157.56 nm to 1191.83 nm, with a significant red shift. According to Equations (4) and (5), we could get the sensitivity *S* = 342.7 nm/RIU, *FOM* = 199.2. Under higher light confinement conditions, the LSPRs (LSPRs, the local surface plasmon resonances) excited at the silver nanostructure/spacer interface are more strongly coupled to the SPR excited at the silver mirror/SiO_2_ spacer interface, thereby reducing absorption. These features provide ample theoretical guidance for designing high performance sensors.

## 4. Conclusions

In conclusion, comprehensively, we have proved the absorption of monolayer graphene structure is achieved through using the critical coupling theory and coated photonic crystal plate (SiO_2_) on the super-thick metal reflector. By changing the thickness of SiO_2_ (*d*_2_) and the thickness of PMMA (*d*_4_), the system experiences three states about under-coupling, critical coupling, and over-coupling because of the leakage rate of resonance. The critical coupling of guide mode resonance is mainly related to the ratio between the radii of the stomatal of the elliptic cylinder air hole and the period of the structure. When the definite ratio is obtained, the spectral position of absorption peak can be adjusted through altering the radii and structural period of the stomata about the elliptical cylindrical air hole. The structure has good sensing performance *S* = 342.7 nm/RIU, *FOM* = 199.2. Through studying the refractive index of the medium, which is realized that it has good development potential in biosensors. The proposed structure is simple and the program is versatile, which provides an effective method for improving the interaction of light materials for atomic thin two-dimensional materials in the future. Our results will play an important role in graphene-based photoluminescence, biosensors, which also can be used to design and manufacture photodetectors, chemical sensors, optical switches, modulators, and many other optoelectronic devices.

## Figures and Tables

**Table 1:** Comparison results of presented absorber with other similar monolayer graphene absorbers.

Reference	Wavelength/nm	FWHM/nm	Q
[33]	1500	20	75
[41]	1500	14	107.1
[46]	1526.5	18	84.8
[47]	1554	2.5	621.6
[48]	1556	150	10.37
[49]	1320	/	170
Present	1022.9	1.5	664.2
